# The Surface Characteristics, Microstructure and Mechanical Properties of PEEK Printed by Fused Deposition Modeling with Different Raster Angles

**DOI:** 10.3390/polym14010077

**Published:** 2021-12-26

**Authors:** Sasa Gao, Ruijuan Liu, Hua Xin, Haitao Liang, Yunfei Wang, Junhong Jia

**Affiliations:** 1 College of Mechanical & Electrical Engineering, Shaanxi University of Science & Technology, Xi’an 710021, China; gaosasa@sust.edu.cn (S.G.); mememoi@163.com (R.L.); 1905031@sust.edu.cn (H.L.); wang17809163790@163.com (Y.W.); jhjia@sust.edu.cn (J.J.); 2 State Key Laboratory of Applied Optics, Changchun Institute of Optics, Fine Mechanics and Physics, Chinese Academy of Sciences, Changchun 130033, China; 3 Tribology Research Institute, School of Mechanical Engineering, Southwest Jiaotong University, Chengdu 610031, China

**Keywords:** surface characteristics, mechanical properties, fused deposition modeling (FDM), polyether-ether-ketone (PEEK), raster angle

## Abstract

Additive manufacturing provides a novel and robust way to prepare medical product with anatomic matched geometry and tailored mechanical performance. In this study, the surface characteristics, microstructure, and mechanical properties of fused deposition modeling (FDM) prepared polyether-ether-ketone (PEEK) were systematically studied. During the FDM process, the crystal unit cell and thermal attribute of PEEK material remained unchanged, whereas the surface layer generally became more hydrophilic with an obvious reduction in surface hardness. Raster angle has a significant effect on the mechanical strength but not on the failure mechanism. In practice, FDM fabricated PEEK acted more like a laminate rather than a unified structure. Its main failure mechanism was correlated to the internal voids. The results show that horizontal infill orientation with 30° raster angle is promising for a better comprehensive mechanical performance, and the corresponding tensile, flexural, and shear strengths are (76.5 ± 1.4) MPa, (149.7 ± 3.0) MPa, and (55.5 ± 1.8) MPa, respectively. The findings of this study provide guidelines for FDM-PEEK to enable its realization in applications such as orthopedic implants.

## 1. Introduction

Polyether-ether-ketone (PEEK) is a potential biomaterial that could replace traditional metal or ceramic parts for biomedical applications due to its excellent biocompatibility and desirable mechanical properties [1,2,3,4,5]. Compared with traditional injection molding and extrusion technology, additive manufacturing (AM) offers a number of advantages for designing and manufacturing customized and complex functional parts with greater flexibility and low manufacturing cost [6,7,8]. Among all the AM manufacturing methods, Gao W et al. reported that fused deposition modeling (FDM) is the most commonly used and low-cost 3D printing technology for thermoplastic materials, which has been an alternative method to process PEEK parts [9,10]. However, a number of challenges still exist in successfully realizing FDM printed PEEK owing to its high melting temperature, great melting expansion, and especially its microstructural packing state [11]. Currently, the influence of printing parameters on the formability and mechanical properties are attracting more and more interest, which should be investigated thoroughly to enlarge the biomedical applications of FDM printed PEEK.

In order to investigate processing PEEK on a 3D printer for thermoplastic modelling, Valentan et al. developed a new FDM machine to produce PEEK medical implants, and the mechanical properties of manufactured products were investigated. The results showed that the strength of the FDM-PEEK samples was approximately half of the tensile strength of molded PEEK [12]. Vaezi et al. expressed that thermal conditions (e.g., extrusion temperature and ambient temperature) need to be carefully controlled in order to ensure good interlayer bonding and to minimize warpage and delamination [13]. Because the temperature fluctuations directly affected interlayer bonding, Kumar et al. investigated the effect of process parameters (chamber temperature, bed temperature, screw speed, deposition speed, standoff distance between nozzles, and bed surface) on layer bonding, layer thickness, and width during the fused layer modeling process [14]. Wu et al. showed that the chamber temperature has more influence on the warping deformation of FDM-PEEK samples than that the nozzle temperature does, and the warping deformation of FDM-PEEK samples reduces with increasing chamber temperature, whereas it has the tendency of a parabola going upwards with increasing nozzle temperature [15]. Moreover, the research from Hu and his colleagues show that the uniformity of temperature field during FDM printing was essential to ensure the high mechanical performance of PEEK [16], and a heat controller could be used to monitor the extrusion temperature.The melting condition and fluidity of PEEK during FDM fabrication were investigated by employing finite element analysis [17]. The parameters of a heating temperature of 440 °C, printing speed of 20 mm/s, and printing layer thickness of 0.1 mm are recommended to reduce the internal defects and improve the bonding strength and surface finish. Yang et al. investigated the relationship between various thermal processing conditions (the ambient temperature, the nozzle temperature, and heat treatment methods) in the FDM process and crystallinity and mechanical properties (tensile strength, elastic modulus, and breaking elongation) of pure PEEK material [18].

Apart from above noted printing parameters, the mechanical performances of PEEK products are significantly affected by the infill ratio, building orientation, and raster angle [19,20,21]. Micro-CT scan confirmed that under 100% infill ratio, there are still some internal voids presented, regardless of the applied building orientation [20]. In term of macroscopic mechanical performance, the consistency of printing direction and loading direction is important [21]. Wu et al. studied the influence of layer thickness (200, 300, and 400 μm) and raster angle (0°, 30°, and 45°) on the mechanical properties (tensile, compressive, and bending strengths) of FDM printed PEEK [22]. The results expressed that the optimal mechanical properties of PEEK were found in samples with a 300 μm layer-up thickness and a raster angle of 0°/90° while only focusing on the tensile strength and bending strength, which is not sufficient to evaluate the comprehensive performance of PEEK. All of these studies have significantly contributed to understanding the effects of temperature on the design, control, and realization of FDM printing PEEK, some of which even investigated the printing parameters on the mechanical performance of PEEK printed parts.

In addition to the mechanical performance, the biocompatibility of FDM printed PEEK has been studied in different in-vitro cell culture experiments [23,24]. Significant increases in cell adhesion, metabolic activity, and proliferation have been observed after 5 days exposure in an osteoblast cell line [23]. Moreover, Zhao had stated that no cytotoxic products were found during FDM-PEEK fabrication [24]. These preliminary findings are favorable for the adoption of FDM-PEEK in the orthopedic field, but long-term animal trials are still needed. When PEEK is applied as an implant, appropriate surface layer characteristics and surface finish are essential. For instance, surface roughness and wettability are closely related to the cell attachment and protein absorption, which in turn affect the overall host tissue response [25]. In addition, surface layer hardness is a key index of wear resistance, which indirectly determines the service life of an artificial joint [26]. However, little information exists on surface characteristics of FDM printed PEEK.

The aforementioned research mainly focused on investigating the influence of FDM process parameters (ambient temperature, nozzle temperature, printing speed, printing layer thickness, etc.) on the mechanical properties of PEEK printed parts. However, current research on the mechanical properties, microstructure, and surface quality of PEEK parts is insufficient. There is a lack of research on the shear strength and surface characteristics of FDM-PEEK, in particular; the basic failure mechanism is presented with little detail. In the current study, the mechanical properties (tensile, flexural, and shear) and surface layer attributes (wettability, hardness, and roughness) of FDM-PEEK were systematically evaluated. Series of mechanical tests were performed, followed by appropriate material examination and surface layer characterization to investigate the influences of the FDM process on the micro-structure and failure mechanism of PEEK. The potential effects of raster angle on the bulk mechanical strength and surface layer attributes of PEEK product were investigated as well.

## 2. Experiential Methods

**Figure 1:**
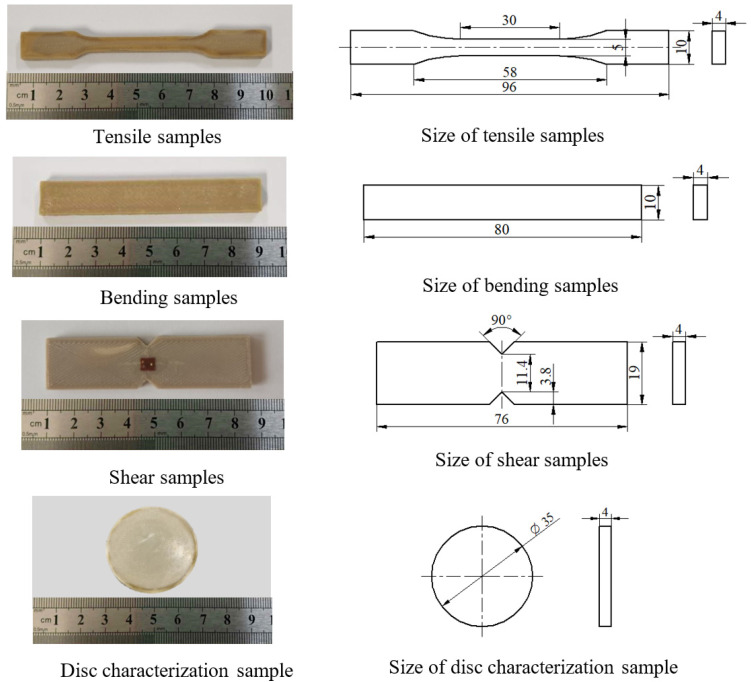
The shapes and dimensions of tensile samples, bending samples, shear samples, and disc characterization samples.

Test samples were manufactured by a P220 FDM printer (Apium Additive Technologies GmbH, Karlsruhe, Germany) using PEEK filaments (Apium^®^ PEEK 450 Natural) with a diameter of 1.75 mm. The printing accuracy of the printer is ±0.05 mm. Sample geometries were produced in the x-y plane based on the corresponding standard of each test described below (for tensile testing samples ISO527-2 (2012); for flexural testing samples ISO178 (2010); for shear testing samples ASTM-D5379M (2012); and the disc samples standard is based on the fixture of the friction-abrasion testing machine), as shown in Figure 1, which allowed us to evaluate the mechanical properties of the samples against existing data for injection molded PEEK. The adopted FDM processing conditions and parameters used for PEEK specimens in this research are provided in Table 1.

**Figure 2:**
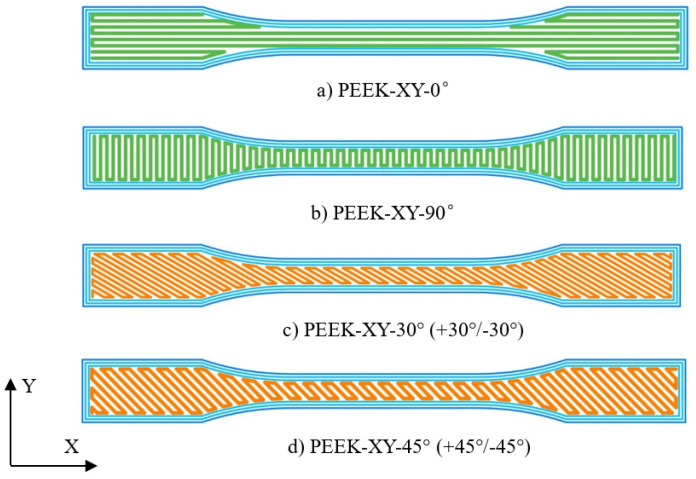
The schematic diagram of the printing path with different raster angles for tensile samples.

Figure 2 gives the FDM configurations for tension specimens with four different raster angles (PEEK-XY-0°, PEEK-XY-90°, PEEK-XY-30°, and PEEK-XY-45°). In this study, raster angle 0° denotes the printing path is along the X direction (horizontal direction), and raster angle of 90° indicates the printing path is along the Y direction (vertical direction). The printing path is the same for all the layers of specimens with raster angle 0° (named as PEEK-XY-0°) and raster angle 90° (named as PEEK-XY-90°). In contrast, raster angle 30° (named PEEK-XY-30°) stands for +30°/−30° cross angle printing, which means one layer’s printing path is +30° to the X direction and subsequent layer’s printing path is −30° to the X direction, and this loop iterates until the printing ends. Printing path of PEEK-XY-45° follows the same definition as PEEK-XY-30°. Only one shell consisting of three filaments (approximately 1.2 mm width) was used to create the outline contour. Subsequently, 100% infill was applied to fabricate testing specimens. The final dimension accuracy of the FDM printed testing specimen is ±0.1 mm. Five specimens for each raster angle are prepared, which are the same for other tests. Prior to surface inspection, disc samples were rinsed twice in distilled water and ultrasonically cleaned in a propan-2-ol bath for 20 min. Finally, they were wiped with acetone and placed in a dust-free container to dry naturally [27]. In this research, one-way ANOVA with pair-wise multiple comparison was adopted to compare the results. All the statistical analysis was performed using Sigma-plot Version 11.0 (Systat Software Inc., Palo Alto, CA, USA), the significance level was set at *p* < 0.05, and an error bar was used to represent the standard deviation. Moreover, injection molded PEEK parts were selected as a comparison using the same PEEK 450G as the FDM printed PEEK.

### 2.1. Physical Properties

#### 2.1.1. Micro-Structure and Thermal Properties

Micro-structural analysis was conducted with the intention to study the influence of the FDM fabrication process on the crystal unit cell structure of PEEK polymer. A D/max 2200PC XRD instrument (Rigaku Corporation, Tokyo, Japan) with a CuKα radiation source of 1.524 Å was utilized. The working voltage and current were 40 kV and 40 mA, respectively. A scan range of 5–50° and a step of 0.02° were adopted.

The thermal properties of FDM printed PEEK parts are determined by a differential scanning calorimeter (DSC-1, Mettler Toledo, Columbus, OH). A scan rate of 50 °C·min^−1^ was chosen in order to minimize the effect of molecular reorganization and recrystallization [28]. Only one heating scan was conducted, and the obtained thermal graphs were used to determine the thermal attributes of PEEK 450G and FDM fabricated PEEK.

#### 2.1.2. Crystallinity

From the collected thermograms obtained by DSC, the peak area between 200 and 400 °C was used to calculate the bulk crystallinity, according to Equation (1).

(1) $$X_{c}=\frac{\Delta H_{f}}{\Delta H_{c}}\times 100\%$$

where *X_c_* is the crystallinity of PEEK, Δ*H*_f_ is the heat of fusion, and Δ*H*_c_ is the theoretical heat of fusion of 100% crystalline PEEK (130 J/g) [29].

Non-destructive Raman spectroscopy (DXR, Thermo Scientific, Madison, WI, USA) was then used to indirectly measure the surface layer crystallinity of FDM fabricated PEEK. A helium-neon 532 nm laser source and a 900 line·mm^−1^ holographic grating were used. Forty examination points were evenly assigned across the specimen. The peak intensity ratio between the C–O–C group (1146 cm^−1^) and the phenol ring (1598 cm^−1^) was used as an indication [30].

#### 2.1.3. Surface Characteristics (Surface Roughness, Hardness, and Wettability)

Surface roughness was measured using a contact typed roughness measuring device (Mar Surf M 300C, Mahr GmbH, Göttingen, Germany) with a 2 μm radius diamond stylus and an examining area of 4 × 4 mm^2^. A Gaussian filter (cut-off length 0.8 mm) was used to separate roughness from waviness [31]. Special attention should be paid to avoid the scratching of the surface when using the contact typed roughness measuring device. If the experimental conditions allow, a non-contact technique such as the AFM and optical profilometry are suggested to be used for surface roughness measuring. The surface layer hardness was measured using a shore-D hardness tester (LXD-A, SHSIWI Ltd., Shanghai, China), and ten examination points were randomly selected.

With the intention to quantify the surface wettability of FMD fabricated PEEK, the static contact angle and surface energy were determined with respect to distilled water at room temperature. A contact angle instrument (OCA20, Dataphyscs, Filderstadt, Germany) was used and 20 examination points were randomly taken from the surface of the disc sample.

### 2.2. Mechanical Tests

The tensile, flexural, fractural, and shear responses of FDM printed PEEK were investigated following the appropriate ISO guidelines for polymeric materials.

#### 2.2.1. Tensile Testing

According to ISO527-2 (2012) [32], PT-1036PC universal testing machine (Baoda Ltd., Guangzhou, China) was used for tensile test at a strain rate of 1%· min^−1^ under an ambient temperature of 20 °C to compare tensile behavior of FDM printed PEEK with other available AM materials and techniques. A type 1BA specimen was chosen and the gauge length was 25 mm; each of the specimen configurations was tested at least three times to assess repeatability. After tensile tests, the micromorphology of fractured printed samples was observed using scanning electron microscopy (Verios 460 SEM, FEI, Hillsboro, OR, USA) to investigate the influence of raster angle on the fracture mechanism of FDM fabricated PEEK. As the fracture mechanism is always closely related to its internal defect, the gauge section of tensile specimen was examined via Micro-CT scanning (Y.Cheetah, Feinfocus, Hamburg, Germany) to observe its internal defect, which can help to explain the basic failure mechanism of PEEK specimens fabricated by FDM.

#### 2.2.2. Flexural Testing

The flexural test specimens were printed at the size of 80 × 10 × 4 mm^3^ with four different raster angles. Three-point flexural tests were conducted according to ISO178 (2010) standard procedure on a PT-1036PC universal testing machine at a constant crosshead speed of 1 mm·min^−1^ to compare the bending behavior (flexural strengthen) of four different raster angles of FDM-PEEK [33].

#### 2.2.3. Shear Testing

Shear test was conducted according to ASTM-D5379M (2012) [34] on the Instron 8801 fatigue testing machine (Instron Ltd., Norwood, MA, USA) at a loading rate of 2 mm·min^−1^. Standard v-notched beam test coupons were fabricated by FDM with four different raster angles (PEEK-XY-0°, PEEK-XY-30°, PEEK-XY-45°, and PEEK-XY-90°); the test specimens were then loaded to failure. Each specimen configuration was tested at least three times to ensure the test consistency, and loading force and shear strain were recorded throughout the test.

## 3. Results and Discussion

### 3.1. Physical Properties

#### 3.1.1. Micro-Structure and Thermal Properties

**Figure 3:**
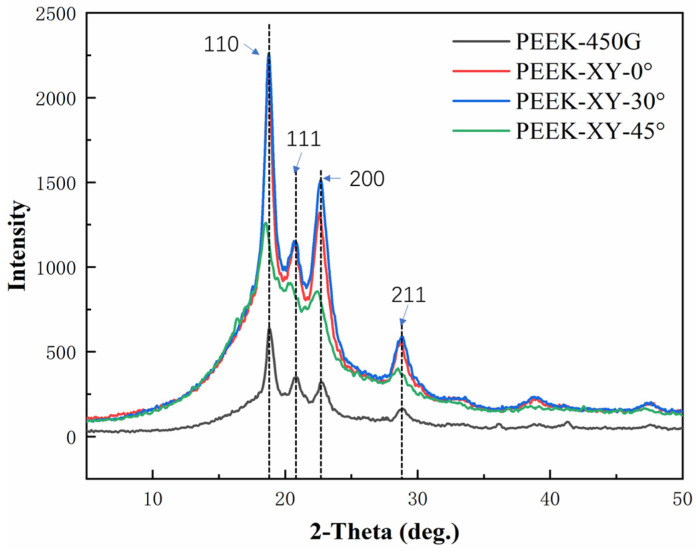
XRD scans of PEEK 450G and FDM printed PEEK parts.

**Figure 4:**
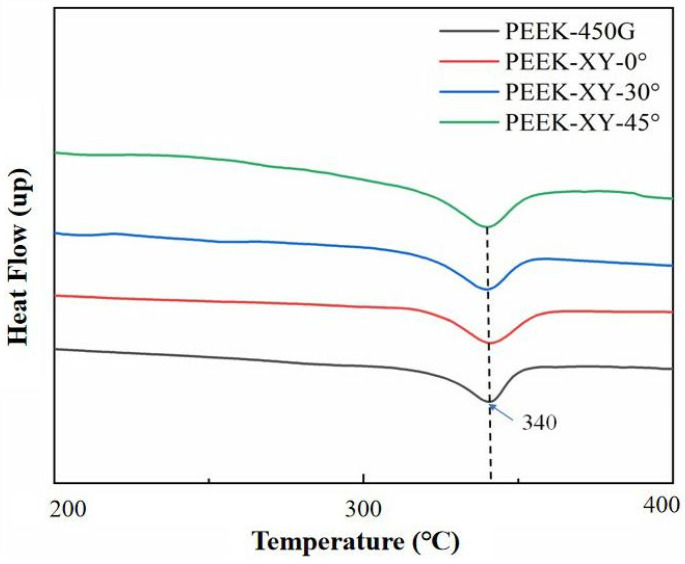
DSC thermograms of PEEK 450G and FDM printed PEEK parts.

It is well documented that the polymer backbone of PEEK exhibits a zigzag spatial configuration, and its crystal unit cell has an orthorhombic structure [35]. From Figure 3, it is obvious that the diffraction patterns of FDM printed PEEK parts were similar with that of injection molded PEEK (i.e., PEEK 450G). Visible diffraction peaks were observed at 2θ (around 19°, 21°, 23°, and 29°), which corresponded to the 110, 111, 200, and 211 planes, respectively. In addition, the obtained thermograms, which were taken from the first heating scan, were similar among PEEK 450G and FDM printed PEEK parts. As shown in Figure 4, a single melting endotherm (near 340 °C) was always seen and no recrystallization exotherm was observed. For PEEK 450G, the obtained DSC results are in good compliance with the literature findings [35,36]. According to the XDR and DSC analysis, we can conclude that the micro-structure and thermal attributes of PEEK material were not altered during the FDM fabrication process.

#### 3.1.2. Crystallinity

The bulk crystallinities of FDM fabricated PEEK were calculated to range from 23.53% to 27.76%. These values are relative smaller than those of the injection molded PEEK 450G (reported as from 31.9% to 40.5%) [36]. However, the obtained I_1146_/I_1598_ ratios (as listed in Table 2) indicate FDM fabrication did not lead to significant alteration in the surface layer crystallinity of PEEK. The differences in bulk and surface layer crystallinity results are likely due to the skin and core effect. In addition, the reduction in bulk crystallinity is probably caused by the utilization of a relative lower bed temperature [18]. PEEK is a linear semi-crystalline thermoplastic, and its mechanical strength is mainly contributed by the crystal phase [37]. The reduction in bulk crystallinity may lead to deterioration in the mechanical performance. Additional post-fabrication thermal treatment can be used to overcome this issue.

#### 3.1.3. Surface Characteristics

**Figure 5:**
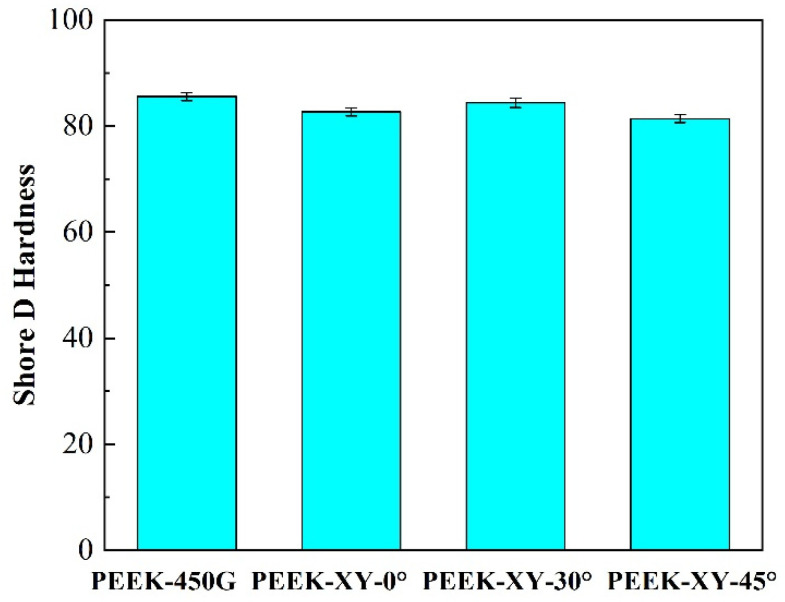
Surface hardness of PEEK 450G and FDM fabricated PEEK parts.

Hardness is not only the comprehensive mechanical property index of material, but also the most important factor affecting the wear resistance of material. As shown in Figure 5, the surface layer hardness of PEEK was significant reduced (*p* ≤ 0.028) when applying FDM. The deterioration of surface mechanical properties may lead to excessive wear and cause premature failure of artificial joint prosthesis. A detailed comparative tribological investigation is needed to further assess the wear potential of FDM fabricated PEEK.

As listed in Table 3, the initial surface roughness of FDM printed PEEK parts were in sub-micro level (*Ra* from 0.613 to 0.667 μm) and met the basic surface quality requirement of polymeric prothesis. For NuNec^®^ PEEK self-mating disc prothesis prepared by injection molding and machining, its surface roughness was documented as *Ra* ≤ 0.585 μm [31]. After polishing treatment, *Ra* can be further reduced to the range of 0.106 to 0.155 μm. This result agreed well with other literature using the same P220 FDM printer. The reported *Ra* surface roughness of the polished PEEK sample was (0.17 ± 0.08) μm [23].

**Figure 6:**
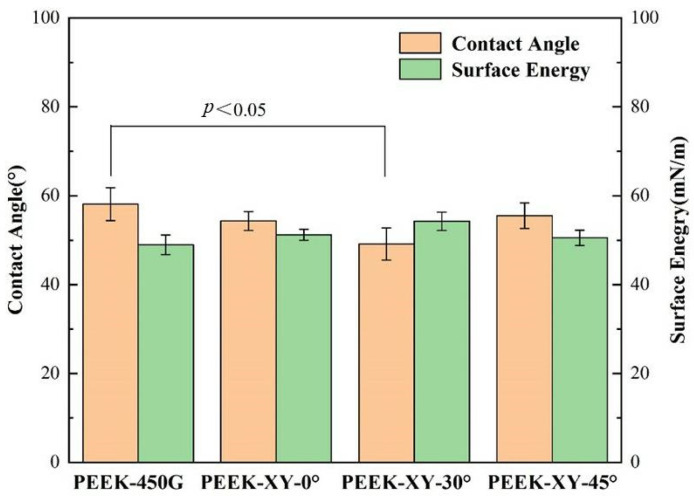
Contact angles and surface energy of PEEK 450G and FDM fabricated PEEK parts.

The contact angle measurements of PEEK 450G and FDM printed PEEK parts are shown in Figure 6. Apart from PEEK-XY-30°, there was no significant alteration in contact angle when adopting FDM fabrication. PEEK-XY-30° became more hydrophilic than that of injection molded PEEK 450G. Among the three different raster angles, XY-30° led to the smallest contact angle with the highest surface energy, which was favorable for cellular adhesion. For a successful artificial implant, adequate wettability is essential for ensuring good host tissue response. The surface layer should not be either extremely hydrophobic or hydrophilic so it is conducive to protein absorption and reorientation [38].

### 3.2. Mechanical Properties

#### 3.2.1. Tensile Testing

**Figure 7:**
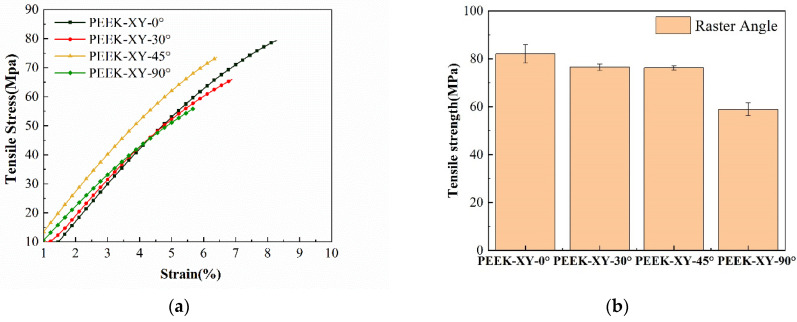
Tensile behaviors with varied raster angles. (**a**) Tensile stress-strain curves; (**b**) average tensile strengths against raster angle.

The tensile stress-strain curves of PEEK specimens with varied raster angles are depicted in Figure 7. It was obvious that PEEK specimens only exhibited linear elastic deformation, regardless of the raster angle. PEEK-XY-0° has the highest tensile strength value of (82.0 ± 3.8) MPa, which is roughly about 82% of that of the injection molded PEEK 450G (i.e., 100 MPa). In comparison, PEEK-XY-90° possesses the lowest tensile strength of (58.9 ± 2.7) MPa, which is significantly smaller than other groups (*p* < 0.01). PEEK-XY-30° and PEEK-XY-45° exhibit moderate tensile strengths, (76.5 ± 1.4) MPa and (76.2 ± 0.9) MPa, with no statistical difference. It can be concluded that raster angles have an important impact on the tensile performance of PEEK specimens. This finding is in accordance with the published literature [20,21]. In practice, FDM fabricated PEEK acts as a laminated structure rather than a unified structure. The maximum sustainable force is along the filament infill direction, whereas the bonding strength between the filaments is weak.

**Figure 8:**
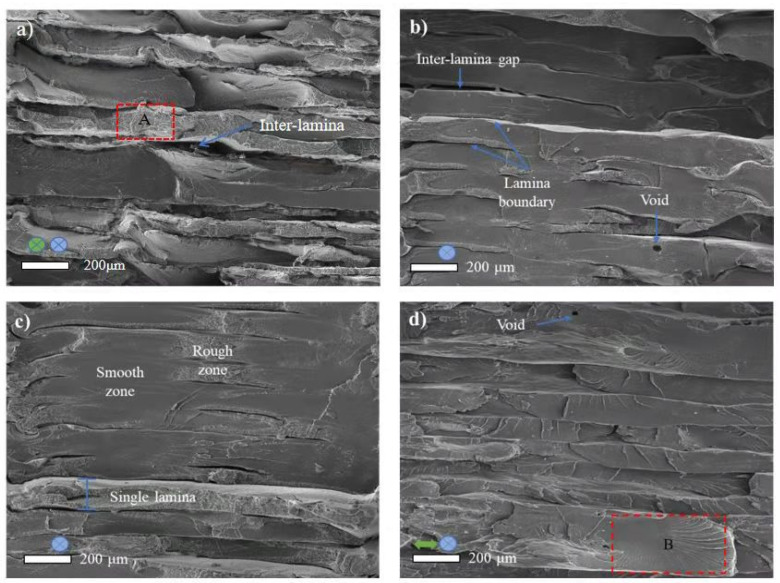
SEM pictures of the cross-section of fractured tensile specimens. (**a**) PEEK-XY-0°; (**b**) PEEK-XY-30°; (**c**) PEEK-XY-45°; (**d**) PEEK-XY-90°. Green arrows and circles indicate the fabrication direction; blue circle indicates the loading direction.

To further study the influence of raster angle on the fracture mechanism of FDM fabricated PEEK, SEM fractography was performed. The cross-section views of fractured tensile specimens are shown in Figure 8. Stratification-like appearance with a clear lamellar boundary was seen in each group. Moreover, inter-lamina gaps and intra-lamina voids were the common features. Among the four groups, PEEK-XY-0° was relatively rougher and uneven (Figure 8a). This was mainly because the direction of tensile loading was parallel to the raster angle, thus the infilled PEEK filament was under uniaxial loading with a larger strain (refer to Figure 7). For PEEK-XY-90°, the direction of tensile loading was perpendicular to the raster angle, thus the external force was sustained only by the weak inter-lamina bonding strengths.

**Figure 9:**
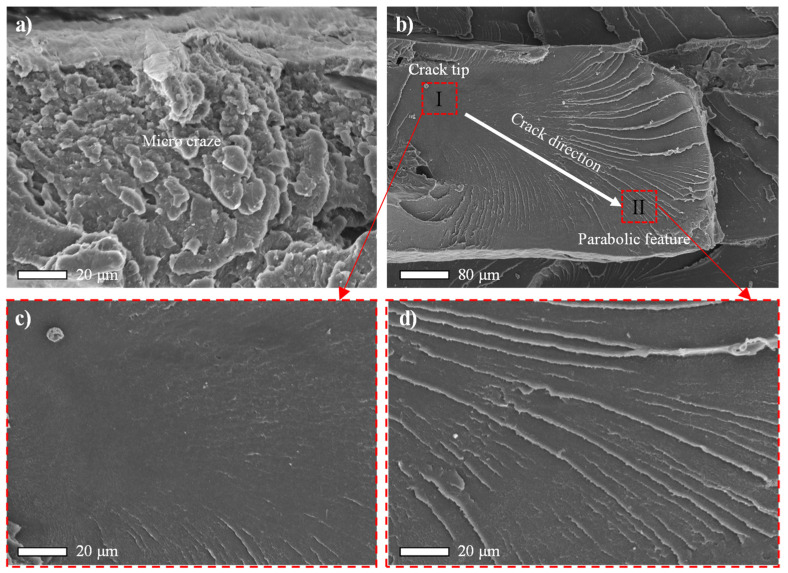
SEM pictures of fracture mechanism. (**a**) The magnified view of area ‘A’ in Figure 8a; (**b**) the magnified view of area ‘B’ in Figure 8d; (**c**) the magnified view of crack tip region ‘I’; (**d**) the magnified view of parabolic crack growth region ‘II’. White arrow indicates the crack propagation direction.

Despite the differences in adopted raster angle, the basic fracture mechanism of each group was still the same. As shown in Figure 9, cracks initiated and propagated along the fracture direction, until they reached rapid fracture zone. Moreover, the parabolic-like feature (Figure 9c) indicated the growth of a crack. This kind of fracture mechanism was also reported by Wang and his colleagues [17] when studying the tensile mechanical performance of PEEK prepared by a customized FDM printer. In contrast with the PEEK 450G [17,37], void nucleation was not observed in FDM fabricated PEEK. Cracks may originate from the internal defect related stress concentration site. Void, as one type of internal defect, is commonly found in PEEK studies using either FDM [19,39] or SLS [40] techniques. In practice, the fracture mechanism obeyed by PEEK is always closely related to its internal defect.

**Figure 10:**
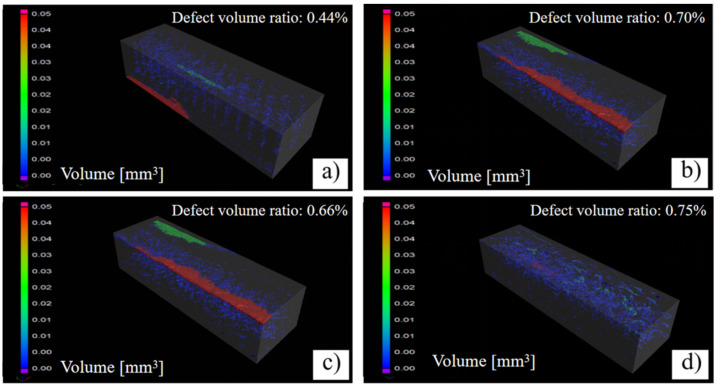
Micro-CT scans of the gauge section of tensile specimens with different raster angle. (**a**) PEEK-XY-0°; (**b**) PEEK-XY-30°; (**c**) PEEK-XY-45°; (**d**) PEEK-XY-90°.

In this study, 100% infill ratio was used to produce compact tensile specimens. However, according to the CT scan results (Figure 10), internal defects (i.e., gap and void) always present. In term of volume content, they were generally less than 1%; XY-0° raster angle resulted in the minimum defect content (0.44 vol.%). The sizes of the defects were observed in the range of 0 to 0.05 mm^3^, and the majority of them were less than 0.01 mm^3^. Moreover, lager defects were always found near the bottom and top surface layers of the specimen. This may be due to the large thermal mismatch during PEEK filament deposition [20]. Post-treatment (e.g., hot isostatic pressing) can be used to densify FDM fabricated PEEK, and thus improve its mechanical strength.

#### 3.2.2. Flexural Testing

**Figure 11:**
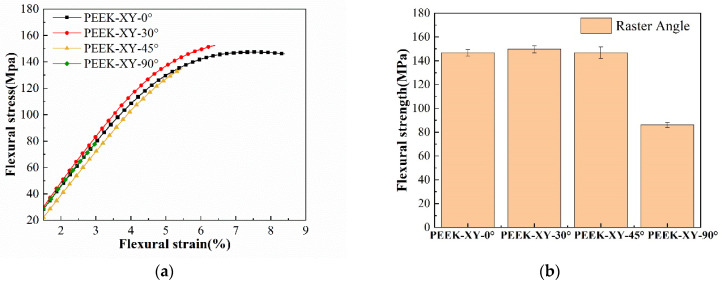
Flexural behaviors with varied raster angles. (**a**) Flexural stress-strain curves; (**b**) average flexural stress against raster angle.

The obtained three-point bending test results are shown in Figure 11. Among the four raster angles, XY-90° resulted in the lowest flexural strength (86.0 ± 2.1) MPa, which is significantly smaller than that of others (*p* < 0.001). In contrast, there is no significant difference between PEEK-XY-0°, PEEK-XY-30°, and PEEK-XY-45° (*p* ≥ 0.516). The recorded flexural strengths are in the range of 146.8 to 149.7 MPa. This agrees well with other PEEK FDM studies: (142.0 ± 5.6) MPa for XY-0° [21], and relatively higher than that of SLS prepared PEEK ((123.0 ± 2.5) MPa) [40]. In comparison with annealed PEEK 450G ((167.2 ± 7.7) MPa [41]), there is at least a 10% reduction in the flexural strength when using FDM fabrication. During the flexural test, the superior and inferior laminae of PEEK specimens are under contraction and tension, respectively. The impact mechanism of raster angle on the exhibited flexural strength is similar to that of tensile strength.

#### 3.2.3. Shear Testing

**Figure 12:**
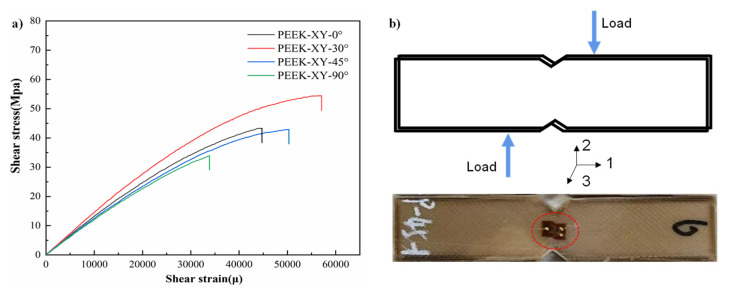
Shear behavior with varied raster angles. (**a**) Shear stress-strain curves; (**b**) picture of v-notched beam test coupon after shear test.

In Figure 12a, the recorded shear stresses are plotted against the shear strains. Raster angle of XY-30°results in the optimal shear performance ((55.5 ± 1.8) MPa), whereas XY-90° leads to the worst ((34.1 ± 1.1) MPa). Raster angles of XY-0° and XY-45° had moderate results and were in between them. As shown in Figure 12b, vertical load is applied to the PEEK laminae during the shear test, which generates shearing force in the G_12_ plane. Stretch-based plastic deformation was visible in the central region of the tested coupon, but no fracture or delamination occurred. This is likely due to the good ductility of the PEEK filament. The in-plane shear force is supported by the week inter-lamina bonding strength. The PEEK coupon is under flexural bending.

According to the mechanical results, it can be concluded that the raster angle does not affect the failure behavior of the FDM fabricated PEEK sample, but only affects the mechanical properties at the macro structural level. The consistency between the infill direction and the direction of external force has a significant impact on the ultimate mechanical strength. Indeed, the mechanical properties of FDM fabricated PEEK still depend on many other factors, such as the filament quality, nozzle diameter, printing speed, printing layer thickness, printing temperature, infill ratio, etc. [17,20,40,42].

## 4. Conclusions

In this paper, a systematic evaluation of FDM printed PEEK parts was conducted in term of micro-structure, surface characteristics, and mechanical properties, in order to investigate the potential effects of raster angle on the macro and micro structural level of PEEK. The results show that the FDM process does not cause alteration in the micro-structure and thermal properties of PEEK material, but does change the bulk crystallinity, which can only reach about 70% of the injection molded PEEK 450G. Surface layer hardness also shows an obvious reduction regardless of the raster angle used. PEEK-XY-30° became more hydrophilic and contributed to the cellular adhesion. Raster angle has shown a significant effect on the mechanical strength, because the FDM-PEEK component is more like a laminated structure, rather than a unified structure. The main fracture behavior is related to the internal defects and the inter-lamina bonding strength. Voids within the PEEK filament can act as stress concentration sites to promote the formation of micro-cracks, which significantly reduce the mechanical strength. The findings of this study can provide guidelines for FDM-PEEK to enable its realization in applications such as orthopedic implants.

## Figures and Tables

**Table 1:** FDM processing parameters provided by manufacturer.

Printing speed	30 mm/s
Nozzle diameter	0.4 mm
Printing temperature	485 °C
Bed temperature	100 °C
Layer thickness	0.1 mm
Infill ratio	100%

**Table 2:** The obtained I_1146_/I_1598_ band ratios.

Samples	Value
PEEK-450G	1.21 ± 0.13
PEEK-XY-0°	1.27 ± 0.28
PEEK-XY-30°	1.16 ± 0.12
PEEK-XY-45°	1.25 ± 0.26

**Table 3:** The surface roughness (*R_a_*) of FDM fabricated PEEK parts, before and after polishing.

Materials	Initial (μm)	Polished (μm)
PEEK-XY-0°	0.63 ± 0.11	0.11 ± 0.03
PEEK-XY-30°	0.67 ± 0.19	0.13 ± 0.03
PEEK-XY-45°	0.61 ± 0.05	0.16 ± 0.02

## Data Availability

The data presented in this study are available on request from the corresponding author.
